# Socioecological factors captured in autism service disparities research on Medicaid-enrolled patients: a rapid evidence review

**DOI:** 10.3389/fpsyt.2026.1767829

**Published:** 2026-03-20

**Authors:** Veronica I. Underwood Carrasco, Sarah Mensah Asantewaa, Katharine E. Zuckerman, Olivia J. Lindly

**Affiliations:** 1 Department of Pediatrics, Oregon Health & Science University, Portland, OR, United States; 2 Department of Epidemiology, OHSU-PSU School of Public Health, Portland, OR, United States; 3 Department of Health Sciences, Northern Arizona University, Flagstaff, AZ, United States

**Keywords:** autism, equity, Medicaid, mental health, review, services

## Abstract

There continue to be disparities in access to quality services that vary from the individual to the policy level. Medicaid is particularly relevant to accessing autism services, since obtaining a diagnosis often confers eligibility, regardless of income requirements, making it a major insurer in this population. Therefore, studies using Medicaid claims data to examine autism service access and utilization may provide unique insights into the demographics being studied and the factors influencing service equity. This rapid evidence review followed the Cochrane Rapid Review Methods Group recommendations and Preferred Reporting Items for Systematic Reviews and Meta-Analysis (PRISMA) guidelines to conduct a literature search across five databases (i.e., ERIC, SCOPUS, PubMed, CINAHL Plus, PsycINFO) to locate articles describing studies of autism services utilization conducted in the United States using Medicaid claims data. Studies had to use Medicaid claims, include an autistic population, be peer-reviewed, original empirical research, and examine variances or disparities in autism services. Case studies or series were excluded. Inclusion criteria were assessed using the Covidence review management tool; 60 articles met all criteria and were included. Information was extracted regarding each study, including characteristics of the study and the included participants, including factors analyzed for variation in service utilization. Results show that existing autism disparities research primarily represents the experiences of school-aged children and young adults (ages 6–21 years), pharmacological and therapeutic services, and individual-level factors, limiting the generalizability of existing research on autism service use.

## 1 Introduction

Autism prevalence has risen steadily, with recent estimates indicating approximately 1 in 31 children in the United States (U.S.) has been diagnosed with autism^1^ (1, 2). Early diagnosis and service utilization play significant roles in optimizing health for autistic children and increasing support for their families (3–5). However, navigating access to autism services can be a lengthy, complex journey that typically starts with diagnostic assessments, often prompted by routine developmental screening or caregiver concerns (6–8). A wide range of services and supports are frequently required following autism diagnoses, including behavioral interventions (i.e., Applied Behavior Analysis), as well as speech-language, physical, occupational, prescription medication, psychiatric, inpatient/hospitalization, acute outpatient, and emergency department services (9–13). Periodic reassessment, service referrals, care coordination, early intervention, special education, parent-mediated intervention, respite care, and financial support may also be needed to help autistic children and their families thrive (14, 15). Unfortunately, disparities remain in access to quality autism services, with persistent variation by intrapersonal, interpersonal, community, structural, and policy-level factors (16, 17).

Medicaid is particularly relevant when investigating U.S. autism disparities, since although primary eligibility requirements are U.S. residence and low to very low income, obtaining a diagnosis often establishes eligibility depending on state laws and available coverage waivers (e.g., diagnosis-based), making it a major insurer for people with autism (11, 13, 17–21). As a result, autistic people are well-represented in Medicaid claims, which are administrative billing data with detailed patient demographics, and diagnostic, procedure, prescription, billing, provider, and location of care information (13, 22). Medicaid claims data can be used to monitor program costs, track service utilization, and inform policy decisions using comprehensive information on eligibility, service use, and payments (11, 22, 23). Though it’s been available since the 1960s, claims data specifically on autism was not made accessible until the 1990s, meaning corresponding prevalence characteristics were only emerging in the early 2000s (13, 16). Yet, claims repositories and databases enable large-scale secondary analysis of service utilization, care patterns, and disparities across populations and states. Moreover, studies using Medicaid claims to examine autism service access and utilization may provide unique insights into who is captured and factors influencing service equity (13, 17).

The purpose of this rapid evidence review was to describe how Medicaid claims have been used in U.S.-based autism research focused on identifying disparities in service access and utilization to assess trends and generalizability. This study advances understanding of autism service disparities by synthesizing factors used to evaluate access to and utilization of autism services in Medicaid claims. Considering that multiple sociological levels impact the access and service experiences of those navigating systems through healthcare, we leverage an adaptation of McLeroy’s socioecological framework embedded into Bronfenbrenner’s ecological model (24, 25) to understand the nested and interconnected influences that add necessary context to systemic barriers and support multilevel, sustainable change. We aimed to assess how studies have examined both individual and intersecting patient characteristics at multiple socioecological levels (i.e., individual, family, provider, neighborhood, system, and historical) to describe patterns and inequities in autism services for Medicaid enrollees.

## 2 Methods

### 2.1 Study design

In January 2024, we began this rapid evidence literature review on secondary analyses of Medicaid claims focused on autism services. A rapid review streamlines or omits certain components of the systematic review process to generate information promptly (26), unlike the traditional comprehensive and explicit methods for identifying, appraising, and analyzing research data (27). We were able to streamline this rapid review by limiting our search to a few databases, excluding grey literature, only including articles freely available and in English, maintaining a single coder scheme after training for final data extraction, and omitting a formal quality assessment or risk of bias. Our interdisciplinary team followed an *a priori* systematic protocol aligned with the Preferred Reporting Items for Systematic Reviews and Meta-Analysis (PRISMA) guidelines. Guidance from Garrity et al. (28) was used to develop our protocol following the Cochrane Rapid Review Methods Group recommendations. Our protocol was preregistered with Open Science Framework (https://osf.io/ftnsa/overview) before the literature search (29). IRB approval was determined unnecessary as we only used public data.

### 2.2 Search strategy

The study team refined research questions around autism diagnosis, service utilization, and cost disparities among Medicaid-insured children with autism in collaboration with health services librarians. The finalized research question—*How have Medicaid claims data been used to study disparities or variations in autism services?*—informed the development of a database-specific literature search strategy co-developed with health services librarians for each target database (i.e., ERIC, SCOPUS, PubMed, CINAHL Plus, PsycINFO). The search strategy aimed to locate articles using Medicaid data on autism with no publication date restrictions; searches were augmented with database-specific thesaurus tools (e.g., ERIC Thesaurus, MeSH terms) to capture related terms to “Medicaid” and “autism.” The finalized literature search strategy was run on January 22, 2024.

### 2.3 Sample

The search identified 1,090 references across all databases. When imported into Covidence’s review management software, these automatically deduplicated to 531 unique references. The study team completed two rounds of pilot testing 10 references, refining inclusion criteria for each phase of review until the average inter-rater agreement reached 80%.

#### 2.3.1 Inclusion criteria

Criteria were developed *a priori* and through pilot testing. Separate process diagrams were used to facilitate coding for each phase of review. During initial cursory review, references were included if the described study was conducted in the U.S., included Medicaid data and an autistic population (i.e., Medicaid-enrolled autistic people), was not a case study or series, and was original empirical peer-reviewed research. A keyword bank included synonyms and commonly used alternative phrases relating to the criteria items. Systematic reviews, literature reviews, and meta-syntheses were included, but scoping and rapid reviews were excluded.

All criteria continued in the subsequent in-depth review, with the additional requirement of examining variances, differences, or disparities in autism services. ‘Services’ was operationalized as diagnosis, cost, medications/prescriptions, insurance enrollment, routine/preventative care, foster care, and mental healthcare, including for concurrent conditions. Studies about autism risk factors and prevalence were excluded.

#### 2.3.2 Article review

**Figure 1:**
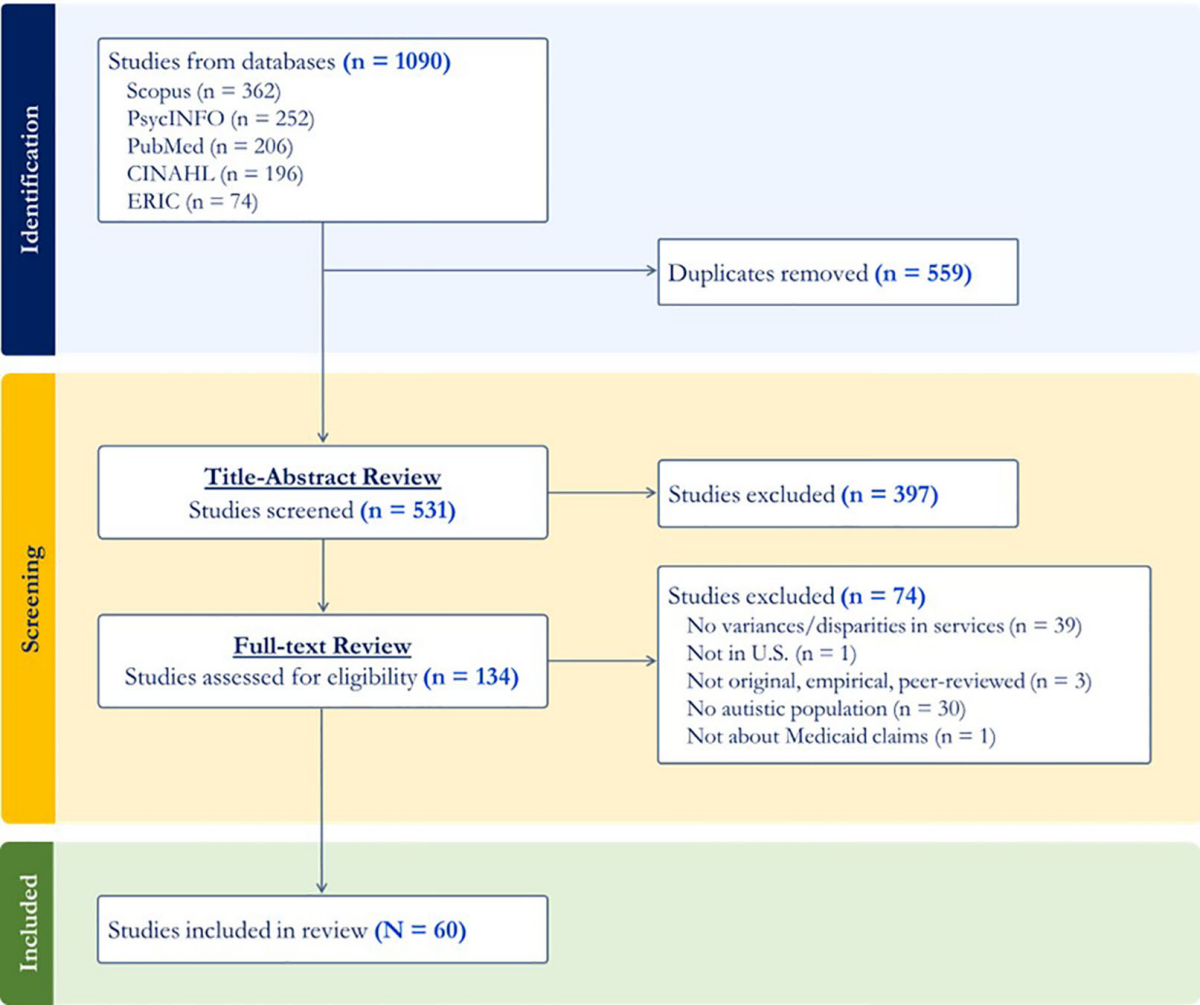
PRISMA Flowchart.

We used a dual data extractor (“coder”) scheme and began with title-abstract screening. Each reference’s title, publication details, and abstract were assessed for relevance against the inclusion criteria, and coders were encouraged to mark articles as “Maybe” if criteria were plausibly applicable. We identified 397 irrelevant references, and 134 proceeded to full-text review. Each reference’s full text was retrieved and assessed in totality for relevance against inclusion criteria; 73 additional articles were excluded. The lead and mentor authors adjudicated coding disagreements throughout piloting and both article review phases. The first two authors validated the preliminary list of included and excluded articles and excluded one additional article. Data were extracted for 60 included studies (**Figure 1**).

### 2.4 Variables and analysis

Questions for data extraction (27 items) characterized article details (i.e., publication year, journal, authors), Medicaid data details (i.e., type of data, years included, source, location, additional data sources), study features (e.g., design, aims, analysis, results, service definition, included services, autism definition, diagnoses definition, socioecological factors), and participant information (i.e., autism and other diagnoses, sample size, race and ethnicity, gender or biological sex, age, language, socioeconomic status). Socioecological factors were operationalized across the individual (i.e., Medicaid enrollee), family and caregiver, provider, organizational (i.e., system delivering services), locale (i.e., location factors, like rurality), and historical level to capture components that influence outcomes from the most micro- (e.g., knowledge, discrimination) to the largest macro-level (e.g., time trends, culture). A codebook described components and requirements for each question and associated responses. The final Qualtrics extraction survey was pilot-tested with five articles (8%) over two rounds until 80% agreement was reached; then, data extraction proceeded independently across three coders, with the codebook iteratively refined throughout. Data validation began in June 2025 to retrieve incomplete data, correct errors, review unclear answers, and reformat categories for analysis, and was completed in December 2025. Descriptive statistics were calculated in Excel.

## 3 Results

### 3.1 Study characteristics

The 60 articles included in this rapid evidence review represent studies from 2002 to 2023 across 26 unique journals (**Table 1**). Most studies were published after 2010 (*n* = 50) with an increasing trend, and the *Journal of Autism and Developmental Disorders* was the most common publishing journal (*n* = 16). The type of Medicaid data most often included was diagnostic codes (*n* = 52) or prescription information (*n* = 24), and most studies used ICD-9 codes (*n* = 49). Medicaid claims from 1993 to 2023 were included, representing data from all 50 states and the District of Columbia (D.C.), though most data used were from 2008 or later. Most studies acquired their data directly from state Medicaid agencies (*n* = 28) or from the Medicaid Analytic eXtract (MAX) dataset (*n* = 26) that includes public insurance claims information covering all states and D.C. from 1999 to 2015. Although 43 (72%) studies included only Medicaid data, 11 studies also included area-level information from federal (e.g., the U.S. Census Bureau) or surveillance databases (e.g., the Autism and Developmental Disabilities Monitoring Network). Included studies were primarily retrospective cohort designs (*n* = 53); the rest were cross-sectional. Statistical approaches and analyses most frequently used regression modeling (e.g., linear or logistic multivariable; *n* = 58).

**Table 1:** Study and participant characteristics from included articles (*N* = 60)^*^.

Study characteristics			
Publication Year (range)		2002-2023	
Claims Years (range)		1993-2018	
Unique Journals (*n*)		26	
		*n* (%)	
Claims Source	State Medicaid agency	28	(45%)
	Medicaid Analytic eXtract (MAX)	26	(43%)
	IBM Market Scan	5	(8%)
	T-MSIS^†^ Analytic Files (TAF)	3	(5%)
	Optum	1	(2%)
	Not Reported	2	(3%)
Participant characteristics		*n* (%)	
Age	Infant: 0–2 years	25	(41%)
	Preschool: 3–5 years	42	(70%)
	School Age: 6–21 years	55	(92%)
	Adult: >21 years	19	(32%)
Sex	Female	55	(92%)
	Male	55	(92%)
	Not Reported	5	(8%)
Race & Ethnicity	White	46	(77%)
	Black or African American	45	(75%)
	Hispanic or Latine	38	(63%)
	Asian	6	(10%)
	American Indian or Alaska Native	6	(10%)
	Multiracial	5	(8%)
	Native Hawaiian or Pacific Islander	1	(2%)
	Other (e.g., “unknown”)	44	(73%)
	Not Reported	10	(17%)
Service Type	Prescription medication	27	(45%)
	Therapy services	26	(43%)
	Diagnostic services	24	(40%)
	Inpatient/hospitalization	23	(38%)
	Outpatient acute care	22	(37%)
	Psychiatric services	15	(25%)
	Emergency department	11	(18%)
	Foster care	6	(10%)
	Preventative care	1	(2%)
	Over-the-counter medication	1	(2%)
	Other (e.g., long-term care management)	17	(28%)
Factors analyzed^‡^		*n (%)*	
Socioecological Levels	Individual	60	(100%)
	Age	55	(92%)
	Sex or gender	52	(87%)
	Race and/or ethnicity	51	(85%)
	Diagnosis or related concerns	30	(50%)
	Socioeconomic status or position	8	(13%)
	Other (e.g., eligibility, comorbidities)	5	(8%)
	Family & Caregiver	15	(25%)
	Socioeconomic status or position	12	(20%)
	Caregiver age, race, or disability	2	(3%)
	Caregiver education	2	(3%)
	Other (e.g., family structure, autistic siblings)	4	(7%)
	Provider	4	(7%)
	Type or discipline (e.g., behavior analyst)	4	(7%)
	Organizational & Regulatory	41	(68%)
	Service structure	28	(47%)
	State law/policy	28	(47%)
	Federal law/policy	6	(10%)
	Administrative structure/staffing	4	(7%)
	Locale & Community	31	(52%)
	Economic (e.g., neighborhood poverty)	22	(37%)
	Community (e.g., state, rural)	20	(33%)
	Health (e.g., provider availability)	12	(20%)
	Educational (e.g., access to school)	4	(7%)
	Historical	3	(5%)
	Time Trends	3	(5%)

*The unit of analysis is the number of articles; therefore, percentages may not add up to 100% due to categories not being mutually exclusive, as well as rounding.

† (T-MSIS), Transformed Medicaid Statistical Information System.

‡ Subfactors included in the codebook under each socioecological level were omitted from this table if none of the articles included analyzed that specific subfactor; low-count categories were also collapsed, if needed.

### 3.2 Participant characteristics

School-aged autistic children and young adults (6 to 21 years) were the most captured population (*n* = 55) across included studies, closely followed by preschool-aged (3 to 5 years) participants (*n* = 42); infants (<3 years) and adults (>21 years) were only represented in 25 and 19 articles, respectively. Although 5 studies did not report on biological sex or gender, females and males were both captured in the remaining 55 included studies. Among studies reporting race and ethnicity (*n* = 50), the most represented categories were White (*n* = 46), Black (*n* = 45), and “other” race and ethnicity categories (*n* = 44), which most often listed the term “other,” severely collapsed categories that spanned several racial and ethnic groups, or “unknown.” Hispanic participants were represented in over half of these studies (*n* = 38), yet American Indian or Alaska Native, Asian (*n* = 6, each), Multiracial (*n* = 5), and Native Hawaiian and Other Pacific Islander (*n* = 1) participants were only present in at most 10%. Though autism was an inclusion criterion, studies primarily included coexisting condition groups (*n* = 43; e.g., Autism & Depression/Anxiety, Autism & Intellectual Disability, Autism & ADHD, etc.) and also looked at allistic- (*n* = 31; e.g., No Autism & Depression, Intellectual Disability Only, etc.) and other condition groups (*n* = 24; e.g., Intellectual Disability, ADHD, etc.). The most common services examined were prescription medications (*n* = 27), therapies (*n* = 26), diagnosis (*n* = 24), and hospitalization (*n* = 23). The therapies examined were typically behavioral (*n* = 17), occupational, or speech therapy (*n* = 10, each). Outpatient acute care and psychiatric services were also assessed in at least a quarter of included articles, though services in the emergency department, foster care, preventative care, over-the-counter medications, and long-term care management were more rarely studied.

### 3.3 Factors analyzed

Measures used to assess service quality and equity had to operationalize factors contributing to autism service disparities. For example, Medicaid policies are a locale-level factor that include autism as a qualifying condition under Medicaid expansion and/or may have autism-specific Medicaid Home and Community-Based Services (HCBS) waivers, affecting service access. Studies explored variation or disparities in autism services primarily at the individual (*n* = 60), organizational (*n* = 41), and locale level (*n* = 31). Some studies also examined family and caregiver (*n* = 15), provider (*n* = 4), and historical factors (*n* = 3). Individual-level factors typically were age (*n* = 55), biological sex or gender (*n* = 52), and race and ethnicity (*n* = 51). None of the included studies analyzed preferred or household language or generational status and level of acculturation (e.g., years in the U.S., citizenship, immigration status). Organizational factors focused on state law and policy (*n* = 28), including Medicaid waiver programs (e.g., HCBS focused on autism) and eligibility criteria for service reimbursement (e.g., income, diagnosis, household characteristics). Studies also assessed service structure (*n* = 28), including family-centered care and service agency responsible for administering Medicaid (e.g., behavioral health divisions or the Department of Education). Similarly, locale factors were primarily economic (*n* = 22) and community-based (*n* = 20), with a focus on state, rurality, and neighborhood opportunity.

### 3.4 Recent trends

Of the 60 included articles across 11 unique journals of nationally representative studies that predominantly used only Medicaid claims data (*n* = 12) from 1999 to 2018, sourced primarily from the MAX (*n* = 10) and state Medicaid agencies (*n* = 6), 17 (28%) were more recent publications (2020 to 2023). Claims were mainly used for diagnostic codes (*n* = 16), procedure information (*n* = 7), and prescription or billing details (*n* = 6, each). Participants included in this subset were most often reported as White, Black (*n* = 17, each), or Hispanic (*n* = 15), female or male (*n* = 17), and school-aged (*n* = 14). The services analyzed were mainly therapies (*n* = 8), prescriptions, and diagnoses (*n* = 6, each). Most recent studies explored variation or disparities in autism services largely at the individual (*n* = 17), organizational (*n* = 12), and locale level (*n* = 10), focusing on factors like enrollee age, race or ethnicity (*n* = 17, each), and sex (*n* = 16), and state law or policies (*n* = 8).

## 4 Discussion

This rapid evidence review is the first to examine how Medicaid claims data have been used to study disparities in service use among U.S. autistic individuals. Results suggest autism service disparities research using Medicaid claims has increased in prevalence over the last two decades. Most of this research was published in autism-specific journals, and though data spanned multiple years, much of the data represented is over a decade old, which may not fully reflect current trends even if publication increases. Most data are from individual agencies, limiting the generalizability of reported results and the capacity to examine state and policy-level drivers of health and access compared to sources like the MAX. Currently published research using Medicaid claims does not seem to commonly include other data sources, meaning the analysis may be missing critical locale factors (e.g., rurality, neighborhood poverty) or interpersonal nuance (e.g., family dynamics, provider-patient interactions) to better capture effects and understand outcomes. Notably, participants were not engaged qualitatively in conjunction with this data to explore their perspectives on what is contributing to the reported disparities.

Participants tended to be ages 3 to 21 years, meaning toddlers and older adults may be underrepresented. Although this is a large age range, and the imbalance means it is likely to be the largest group, regardless, the different context for therapy options is age-specific. Children below age 3 can access services through federal Early Intervention programs, which can be supported through Medicaid, whereas children and young adults ages 3 to 21 may access services through Early Childhood Special Education programs, which can also be billed to Medicaid from educational settings (30). Once over 21, however, services are no longer as centralized, so the impact of HCBS waivers, eligibility criteria, and available insurance options becomes more notable. The main groups being compared continue to be White and Black, and the large representation of “other” reflects the over-collapsing of people into groups that no longer become meaningful for describing the social context of those captured within it, obscuring and erasing nuance and potential differences. The services assessed reflect a large focus on children and young adults’ pharmacological and therapy utilization. Prescription medications were assessed by more than half of the studies, though many autistic individuals do not take medication, and pharmacological treatment is not indicated for core autism features.

Although some studies included macro-level factors that shape health behaviors and service access, most analyses focused on individual-level factors, limiting the amount of context and confounders captured by covariates in something like a regression model. Even so, factors like language and familiarity with the U.S. health and educational system, which have been noted to impact service access (8, 17), were completely ignored in the included studies. Considering individual data without social and historical context can risk misattribution of societal barriers and inequities to interpersonal choices or circumstances, thus placing the onus on the individual rather than the upstream factors that shape inequitable systems and the related structures that drive disparities. Continued work is necessary to capture the discrete number of participants that are represented across demographic characteristics like age, gender, and race and ethnicity, beyond their presence reported across these articles, to understand the magnitude of [under]representation of these groups in autism research using Medicaid claims.

A key strength of this rapid review is the systematic, PRISMA-aligned approach used to synthesize studies using Medicaid claims on autism services, which spans two decades’ worth of information and helps identify service utilization priorities and the socioecological levels assessed to understand service disparities, from the micro-context of the individual to the macro-context of history. However, as a rapid review, several steps of the full systematic review process were streamlined, and we did not conduct a formal assessment of study quality or risk of bias to grade evidence validity. This limits our ability to evaluate the rigor of the included studies and shows the need for future work that expands into a full systematic review or incorporates quality appraisal. Additionally, our focus on Medicaid claims introduces selection bias, systematically excluding individuals who may be underinsured, dually enrolled, or privately insured. Overall, trends from the most recent studies match the overall sample—including data spanning back to the 1990s—albeit with a smaller magnitude in difference between all possible options. Although included articles mostly capture studies published in the last decade, these results do not necessarily focus on the most recent or current trends in Medicaid claims data among autistic populations.

### 4.1 Future research directions

These findings highlight key gaps in autism services research using Medicaid claims, noting outdated data, minimal representation of other insurance options, and limited analyses focusing on family, provider, and historical influences. Additionally, historically underrepresented racial and ethnic groups were often overlooked or grouped into broad categories. Thus, future research is needed to descriptively assess the evidence base of private insurance claims billed for patients with autism diagnoses, as well as for those with multiple or inconsistent insurance, to describe who is captured and how trends are changing for specific populations, as policies change, across the U.S. These gaps can be intentionally addressed by analyzing more recent data, oversampling underrepresented families to better capture their experiences in the literature, and examining broader social and contextual factors to elucidate and reduce inequity in autism care. Integrating Medicaid claims with other data sources or leveraging mixed methods may also provide in-depth insights into the factors impacting access to autism care.

## 5 Conclusion

Medicaid claims data have been used extensively to examine autism-related services and disparities, with a steady increase in publications throughout the two decades of extant research. Studies primarily focus on school-aged children and young adults with autism who are White, Black, or collapsed into an “Other” group, and analyses mainly center on individual characteristics, organizational policies, and locale contextual factors to understand variation in service use. While considerable attention has been given to service utilization patterns and common patient-level disparities, important gaps remain, particularly regarding sociocultural, family, and provider influences on access to autism care as well as the experiences of historically underrepresented sociodemographic groups. Future research is needed to analyze more recent Medicaid claims on autism and descriptively assess the evidence base of who is captured, and how (and why) trends are changing.

## Footnotes

1 Person-first and diagnosis/identity-first language are used interchangeably throughout this manuscript to acknowledge the different preferences in the larger autism community and the terms used in the included articles.
