# Supplementary material for: Socioecological factors captured in autism service disparities research on Medicaid-enrolled patients: a rapid evidence review
Source: Front Psychiatry. 2026 Mar 20;17:1767829. doi: 10.3389/fpsyt.2026.1767829 (PMC13046498; doi:10.3389/fpsyt.2026.1767829)
Supplement: Supplementary file 1 [file Table1.docx]

Supplementary Material

# Supplementary Data

## Search strategies for each database

### PubMed

((child development disorders, pervasive[mh]) OR (asperger*[tiab] OR autis*[tiab] OR (pervasive[ti] AND development*[ti] AND disorder*[ti]))) AND ((centers for medicare and medicaid services, u.s.[mh] OR medicaid[mh]) OR (medicaid[tiab]))

### Scopus (Elsevier)

*Search within Article Title, Abstract, Keywords*

( TITLE-ABS-KEY ( asperger* OR autis* OR ( pervasive PRE/1 development* PRE/1 disorder* ) ) ) AND ( TITLE-ABS-KEY ( medicaid ) )

### CINAHL Plus with Full Text via EBSCOhost

S1 (MH “Child Development Disorders, Pervasive+) OR autis* OR asperger* OR (TI pervasive AND development* AND disorder*)

S2 (MH “Medicaid+”) OR (MH “Centers for Medicare and Medicaid Services, U.S.”) OR Medicaid

S3 S1 AND S2

### APA PsycINFO via EBSCOhost

**(DE (Autism Spectrum Disorders) OR TX (autis* OR asperger*)) AND TX Medicaid**

### ERIC via EBSCOhost

S1 (DE “autism spectrum disorders” OR autis* OR asperger* OR (TI pervasive AND development* AND disorder*))

S2 Medicaid

S3 S1 AND S2

## Table A1. Final Included Studies (n=60)

| **Authors, Publication Year** | **Article Title** |
| --- | --- |
| Mandell et al., 2002 | Race differences in the age at diagnosis among Medicaid-eligible children with autism. |
| Ruble et al., 2005 | Access and service use by children with autism spectrum disorders in Medicaid Managed Care. |
| Mandell et al., 2006 | Medicaid expenditures for children with autistic spectrum disorders: 1994 to 1999. |
| McDermott et al., 2008 | Injury treatment among children with autism or pervasive developmental disorder. |
| Mandell et al., 2008 | Psychotropic medication use among Medicaid-enrolled children with autism spectrum disorders. |
| Rubin et al., 2009 | State variation in psychotropic medication use by foster care children with autism spectrum disorder. |
| Shattuck et al., 2009 | Utilization of a Medicaid-funded intervention for children with autism. |
| Wang & Leslie, 2010 | Health care expenditures for children with autism spectrum disorders in Medicaid. |
| Semansky et al., 2011 | Medicaid's increasing role in treating youths with autism spectrum disorders. |
| Logan et al., 2012 | High prescription drug use and associated costs among Medicaid-eligible children with autism spectrum disorders identified by a population-based surveillance network. |
| Mandell et al., 2012 | The interplay of outpatient services and psychiatric hospitalization among Medicaid enrolled children with autism spectrum disorders. |
| Peacock et al., 2012 | Autism spectrum disorders and health care expenditures: the effects of co-occurring conditions. |
| Stein et al., 2012 | Impact of a private health insurance mandate on public sector autism service use in Pennsylvania. |
| Williams et al., 2012 | Psychotropic medication use in children with autism in the Kentucky Medicaid population. |
| Bilaver & Havlicek, 2013 | Foster Children with Autism Spectrum Disorder: Prevalence, Length of Stay, and Placement Patterns. |
| Cidav et al., 2013 | Age-related variation in health service use and associated expenditures among children with autism. |
| Khanna et al., 2013 | Use and cost of psychotropic drugs among recipients with autism in a state Medicaid fee-for-service programme. |
| Semansky et al., 2013 | How states use Medicaid to fund community-based services to children with autism spectrum disorders. |
| Wang et al., 2013 | Healthcare service use and costs for autism spectrum disorder: a comparison between Medicaid and private insurance. |
| Cidav et al., 2014 | Home- and community-based waivers for children with autism: effects on service use and costs. |
| Logan et al., 2015 | Aberrant Behaviors and Co-occurring Conditions as Predictors of Psychotropic Polypharmacy among Children with Autism Spectrum Disorders. |
| Schubart et al., 2014 | Psychotropic medication trends among children and adolescents with autism spectrum disorder in the Medicaid program. |
| Shea et al., 2014 | Genetic testing and genetic counseling among Medicaid-enrolled children with autism spectrum disorder in 2001 and 2007. |
| Tan et al., 2015 | Psychotropic Medication Adherence among Community-Based Individuals with Developmental Disabilities and Mental Illness. |
| Kang-Yi et al., 2016a | School-Based Behavioral Health Service Use and Expenditures for Children With Autism and Children With Other Disorders. |
| Kang-Yi et al., 2016b | Decline in Medicaid-Funded One-to-One Behavioral Support Use in School as Children Age. |
| Vohra et al., 2016 | Prescription Drug Use and Polypharmacy Among Medicaid-Enrolled Adults with Autism: A Retrospective Cross-Sectional Analysis. |
| Edelsohn et al., 2017 | Trends in Antipsychotic Prescribing in Medicaid-Eligible Youth. |
| Houghton et al., 2017 | Psychiatric comorbidities and use of psychotropic medications in people with autism spectrum disorder in the United States. |
| Locke et al., 2017 | Ethnic Disparities in School-Based Behavioral Health Service Use for Children with Psychiatric Disorders. |
| Vohra et al., 2017 | Comorbidity prevalence, healthcare utilization, and expenditures of Medicaid enrolled adults with autism spectrum disorders. |
| Cidav et al., 2018 | Foster Care Involvement Among Medicaid-Enrolled Children with Autism. |
| Maddox et al., 2018 | Treatment Utilization by Adults with Autism and Co-Occurring Anxiety or Depression. |
| Yingling et al., 2018 | Time-Lag Between Diagnosis of Autism Spectrum Disorder and Onset of Publicly-Funded Early Intensive Behavioral Intervention: Do Race-Ethnicity and Neighborhood Matter? |
| Shea et al., 2018 | Brief Report: Service Use and Associated Expenditures Among Adolescents with Autism Spectrum Disorder Transitioning to Adulthood. |
| Jariwala-Parikh et al., 2019 | Autism Prevalence in the Medicaid Program and Healthcare Utilization and Costs Among Adult Enrollees Diagnosed with Autism. |
| Rubenstein & Bishop, 2019 | Is the Autism Boom Headed for Medicaid? Patterns in the Enrollment of Autistic Adults in Wisconsin Medicaid, 2008-2018. |
| Shea et al., 2019 | Transition-Age Medicaid Coverage for Adolescents With Autism and Adolescents with Intellectual Disability. |
| Yingling & Bell, 2019 | Underutilization of Early Intensive Behavioral Intervention Among 3-Year-Old Children with Autism Spectrum Disorder. |
| Yingling et al., 2019a | Treatment Utilization Trajectories among Children with Autism Spectrum Disorder: Differences by Race-Ethnicity and Neighborhood. |
| Yingling et al., 2019b | Comparing Neighborhoods of Children With Autism Spectrum Disorder in a Medicaid Waiver Program and a State Population, 2007-2015. |
| Benevides et al., 2020 | Emergency department use among young adult Medicare beneficiaries with autism and intellectual disabilities. |
| Robinson et al., 2020 | A Comparison of Health Care Expenditures for Medicaid-Insured Children with Autism Spectrum Disorder and Asthma in an Expanding Accountable Care Organization. |
| Yingling & Bell, 2020 | Utilization of speech-language, occupational and physical therapy by diagnosis of autism spectrum disorder. |
| Benevides et al., 2021 | Racial and ethnic disparities in benefits eligibility and spending among adults on the autism spectrum: A cohort study using the Medicare Medicaid Linked Enrollees Analytic Data Source. |
| Bilaver et al., 2021 | Understanding Racial and Ethnic Disparities in Autism-Related Service Use Among Medicaid-Enrolled Children. |
| Bishop et al., 2021 | Epilepsy in adulthood: Prevalence, incidence, and associated antiepileptic drug use in autistic adults in a state Medicaid system. |
| Dimian et al., 2021 | Delay to Early Intensive Behavioral Intervention and Educational Outcomes for a Medicaid-Enrolled Cohort of Children with Autism. |
| Benevides et al., 2022 | Occupational Therapy Service Delivery Among Medicaid-Enrolled Children and Adults on the Autism Spectrum and With Other Intellectual Disabilities. |
| Franklin et al., 2022 | Inequities in Receipt of the North Carolina Medicaid Waiver Among Individuals with Intellectual Disability or Autism Spectrum Disorder. |
| Liu et al., 2022 | The Association of the Medicaid 1915(c) Home and Community-Based Services Waivers with Emergency Department Utilization among Youth with Autism Spectrum Disorder. |
| Lohr et al., 2022 | Antipsychotic Medications for Low-Income Preschoolers: Long Duration and Psychotropic Medication Polypharmacy. |
| Schott et al., 2022 | COVID-19 risk: Adult Medicaid beneficiaries with autism, intellectual disability, and mental health conditions. |
| Shea et al., 2022 | Medicaid Disruption Among Transition-Age Youth on the Autism Spectrum. |
| Shoaib et al., 2022 | Autism: Comorbidities and Treatment Patterns in the Real World, a Retrospective Cohort Study Among Children, Adolescents and Adults Newly Diagnosed with Autism. |
| Straub et al., 2022 | Neurodevelopmental Disorders Among Publicly or Privately Insured Children in the United States. |
| Carey et al., 2023a | Association Between Medicaid Waivers and Medicaid Disenrollment Among Autistic Adolescents During the Transition to Adulthood. |
| Carey et al., 2023b | Short report on navigating access to care for Medicaid-enrolled autistic youth and young adults: Examining accrual of intellectual disability diagnoses in adolescence. |
| Rast et al., 2023 | Psychotropic Medication Use in Children and Youth with Autism Enrolled in Medicaid. |
| Roux et al., 2023 | Unrealized Cross-System Opportunities to Improve Employment and Employment-Related Services Among Autistic Individuals. |

## Data Extraction Codebook

| # | Question | Instructions | Clarifications |
| --- | --- | --- | --- |
| *General Article Details* | | | |
| 1 | What is the article’s Covidence ID?  *[text field]* | Write out the article’s Covidence ID without the pound sign. | For example, article #3617 in Covidence would be “3617” |
| 2 | What year was the article published?  *[single choice]* | From the list of possible choices, pick out the publication year of this article. | Make sure you are collecting the publication year and not the year of manuscript acceptance. First date printed regardless of if online or in print. |
| 3 | What was the journal where the article was published?  *[single choice]* | From the list of possible choices, pick out the journal where the article was published. | The journal name should be available on the first page and sometimes, as a running header. Make sure you collect the overall journal, not the name of a special edition or article type. |
| 4 | Who are the authors of the study?  *[text field]* | Copy + Paste the author list exactly how they are listed in the article. | Collect all the info on all authors directly from article. |
| *Medicaid Details* | | | |
| 5 | What type of Medicaid claims data was included?  *[select all that apply]* | Based on how the authors describe their data source, please select all the types of Medicaid claims data used in this study. | [ ] **Administrative data**: only use this label if the authors do not specify further.  [ ] **Procedure codes**: identify all items and services within health services categories. Includes Current Procedural Terminology (CPT) and Healthcare Common Procedure Coding System (HCPCS) Codes  [ ] **Diagnostic Codes**: a tool used to group and identify diseases, disorders/diagnosis/disability, symptoms, poisonings, adverse effects of drugs/chemicals, injuries, complaints/concerns, and other reasons or characteristics of a patient/encounter. Includes use of International Classification of Diseases (ICD) codes.  [ ] **Billing/expenditure data**: only use this label if the authors do not specify further.  [ ] **Prescription drug data:** information about the prescription or use of any medication or drug. Includes use of the National Drug Code (NDC) codes  [ ] Uncertain/Unclear (please explain): select this option if it is not clear what the claims data used was, especially if none of the above words are used to describe it. |
| 5a | Please specify what type of diagnostic codes were included by the authors.  *[select all that apply]* | Based on how the authors describe their diagnostic codes and Medicaid data, please select all the types of diagnostic codes used in this study. | [ ] **ICD-9 codes**: the 9^th^ revision of International Classification of Diseases (ICD) codes. Ex. 299.0  [ ] **ICD-10 codes**: the 10^th^ revision of International Classification of Diseases (ICD) codes. Ex. F84.0  [ ] **Other (please specify)**: if the authors do not specify that they used ICD-9 or ICD-10 codes, please Copy+Paste what the authors do share about which diagnostic codes they used, why, and how. Please use the notation “[insert quote here]” p. [insert page number here] |
| 6 | What years of Medicaid claims data were included in the study?  *[form field]* | Based on how the authors describe their data source, please enter the year range of included Medicaid claims data. | Include the range of years of included Medicaid claims. |
| 7 | Where was the Medicaid claims data sourced from?  *[select all that apply]* | Based on how the authors describe their data source, select the location(s) from which the Medicaid claims were collected. | Only note the most specific information shared by the authors. For example, if an article mentions *“data from multiple states (Arizona, Oregon, Indiana) were included”*, then you would select the specific states (i.e., AZ, OR, IN) but not the option for Multiple States (not specified). If the data used was national and also included D.C., select both ALL 50 states (National) and D.C.  [ ] **Each of the 50 States (each individual state)**: select each of the individual states explicitly mentioned by the authors as included in the final dataset analyzed.  [ ] **D.C.**: District of Columbia. If the authors do not explicitly mention including D.C., do not assume it was included.  [ ] **Multiple States (not specified)**: select this option if the authors note that their data was sourced from multiple states but do not give more detailed information about which states they included.  [ ] **ALL 50 states (National)**: only select this option if the authors day they are using a “national” sample or have data from all 50 states.  [ ] **Other (please specify)**: select this option if the geographical/ location information noted by the authors does not fit into the above categories, e.g., territories, commonwealths, military bases, etc. Please Copy+Paste the information the authors share about their data’s location and add additional context as needed. Please use the notation “[insert quote here]” p. [insert page number here] [insert additional context here, if needed]  [ ] **Unclear**: select this option if the location (or part of the location) of the data is somewhat explained in the article, but it is not very clear where the final analyzed data came from. For example, if the authors only share *“Data from 12 states and territories were assessed and excluded if they had high missingness or low quality. Final included states/territories were analyzed”* and do not further specify which states/territories were included, it is unclear what the final included locations were.  [ ] **Not Reported**: only select this option if the authors share no information about the location of their data. |
| 8 | What was the source of the Medicaid claims data used in the study?  *[select all that apply]* | Based on how the authors describe their data source, please select all sources/entities that provided Medicaid claims data for the study. | [ ] **State Medicaid agency**: claims directly from specific state or territory Medicaid systems, or a multi-location Medicaid system like the group MODRN that harmonizes aggregated state Medicaid data for multi-state analyses.  [ ] **All Payer All Claims (APAC) files**: claims from a reporting program containing administrative health care data.  [ ] **Transformed Medicaid Statistical Information System (T-MSIS) Analytic Files (TAF)**: includes child health insurance plan (CHIP) and Medicaid claims  [ ] **Medicaid Analytic eXtract (MAX) files**: a set of person-level data files on Medicaid eligibility, service utilization, and payments.  [ ] **IBM Market scan**: research databases that provide fully integrated, de-identified, individual-level healthcare claims data that can be used to examine health economics and treatment outcomes.  [ ] **Optum**: this is a vendor option to buy claims  [ ] **Other (please specify)**: select this option if the claims source described by the authors does not fall into the above categories. Please Copy+Paste the information the authors share about their claim’s source and add additional context as needed. Please use the notation “[insert quote here]” p. [insert page number here] [insert additional context here, if needed] |
| 9 | Were any other non-Medicaid data sources included in the study?  *[select all that apply]* | Based on how the authors describe their data source, please select any options that best represent the data included in this study. | [ ] **Only Medicaid data was used**: only select this option if the authors do not use any other data besides that captured in Medicaid claims to complete their analyses.  [ ] **Vital statistics data (please describe)**: compiled information and analysis of information from vital events records reported to the state, like birth and death certificates. Please Copy+Paste the information the authors share about their vital statistics data and add additional context as needed. Please use the notation “[insert quote here]” p. [insert page number here] [insert additional context here, if needed]  [ ] **Electronic Health Record (EHR) data (please describe)**: data collected from patient’s health records, like clinic or hospital encounters and patient characteristics. Please Copy+Paste the information the authors share about their EHR data and add additional context as needed. Please use the notation “[insert quote here]” p. [insert page number here] [insert additional context here, if needed]  [ ] **Survey data (please describe)**: only select this option if the authors used a survey to get more information for their analyses. Please Copy+Paste the information the authors share about their survey data and add additional context as needed. Please use the notation “[insert quote here]” p. [insert page number here] [insert additional context here, if needed]  [ ] **Registry data (please describe)**: data collected from an existing collection of information about individuals, usually focused on a specific diagnosis or condition.  Please Copy+Paste the information the authors share about their registry data and add additional context as needed. Please use the notation “[insert quote here]” p. [insert page number here] [insert additional context here, if needed]  [ ] **Cohort data/databases (please describe)**: data used from existing cohort studies, databases, or national/regional datasets, like the National Survey on Children’s Health (NSCH). This includes surveillance programs. Please Copy+Paste the information the authors share about their cohort/database data and add additional context as needed. Please use the notation “[insert quote here]” p. [insert page number here] [insert additional context here, if needed]  [ ] **Unclear**: only select this option if it seems like the authors may have used an additional source of data, but it is not completely clear where that data came from or what type of data it is. For example, if the authors state *“Medicaid claims data was combined with other administrative data to create the final dataset”* and do not provide any additional information, it is not clear what the *“administrative data”* is or where it came from.  [ ] **Other (please specify)**: select this option if the authors used additional data alongside Medicaid claims, but the source described by the authors does not fall into the above categories. Please Copy+Paste the information the authors share about their additional data and add additional context as needed. Please use the notation “[insert quote here]” p. [insert page number here] [insert additional context here, if needed] |
| *Study Details* | | | |
| 10 | What was this article’s study design?  *[select all that apply]* | Based on how the authors describe their methods, please select the study design(s) that best fits how the study was set up. | Be critical of authors saying “longitudinal design”  [ ] **Retrospective cohort**: studying a group of people where their data have already been collected in the past or specific events of interest have already happened. This study type can be longitudinal in its design.  [ ] **Cross-sectional**: an observational study that analyzes data from a population, or a representative subset, at one specific point in time. The data source for a cross-sectional design can have data at multiple time points, but the design is cross-sectional if only one timepoint is analyzed in isolation.  [ ] **Prospective cohort**: studying a group of people to capture data as it unfolds or to track specific events of interest that have yet to happen. This study type can be longitudinal in its design.  [ ] **Other (please specify)**: select this option if the study design described by the authors does not fall into the above categories. Please Copy+Paste the information the authors share about their study’s design and add additional context as needed. Please use the notation “[insert quote here]” p. [insert page number here] [insert additional context here, if needed]  [ ] **Not Reported**: only select this option if the authors share no information about their study’s design. |
| 11 | What was the Research Question(s)/Aim(s)?  *[text field]* | Copy + Paste the study’s research questions and/or project aims. | Please use the notation “[insert quote here]” p. [insert page number here] [insert additional context here, if needed] |
| 12 | What was the article’s statistical approach/analysis?  *[select all that apply]* | Based on how the authors describe their methods and analyses, please select the statistical approaches/ analyses that best fit how the study data was analyzed. | [ ] **model (please describe)**: a mathematical model(s) using statistical assumptions to examine and compare data across variables or covariates like time, race and ethnicity, biological sex, etc. Please Copy+Paste the information the authors share about their model approach and add additional context as needed. Please use the notation “[insert quote here]” p. [insert page number here] [insert additional context here, if needed]  [ ] **machine learning (please describe)**: Using statistical techniques to develop models that can learn from data and make predictions or decisions. Please Copy+Paste the information the authors share about their machine learning approach and add additional context as needed. Please use the notation “[insert quote here]” p. [insert page number here] [insert additional context here, if needed]  [ ] **geospatial analyses (please describe)**: computational analysis that leverages geographic information, spatial data, location data, high-resolution geographic information system (GIS) imagery, computer vision, and other forms of AI to extract structured data and make comparisons and assessments. Please Copy+Paste the information the authors share about their geospatial approach and add additional context as needed. Please use the notation “[insert quote here]” p. [insert page number here] [insert additional context here, if needed]  [ ] **propensity scoring/matching/weighing (please describe)**: statistical methods that reduce bias in observational data by balancing baseline characteristics between groups. Please Copy+Paste the information the authors share about their propensity approach and add additional context as needed. Please use the notation “[insert quote here]” p. [insert page number here] [insert additional context here, if needed]  [ ] **Instrumental variables approaches (please describe)**: a statistical method used to estimate causal relationships between variables when other methods are biased. Please Copy+Paste the information the authors share about their instrument variable approach and add additional context as needed. Please use the notation “[insert quote here]” p. [insert page number here] [insert additional context here, if needed]  [ ] **Other (please specify)**: select this option if the statistical approach described by the authors does not fall into the above categories. Please Copy+Paste the information the authors share about their study’s statistical approach or design and add additional context as needed. Please use the notation “[insert quote here]” p. [insert page number here] [insert additional context here, if needed] |
| 13a | What were the main results that answered their research question(s)?  *[text box]* | Copy + Paste the main results that answer the study's posed research question(s). | Include any point estimates, confidence intervals, effect sizes, percentage points, and standard deviations (etc.) shared by the authors. Please use the notation “[insert quote here]” p. [insert page number here] [insert additional context here, if needed] |
| 13b | What were the disparities/variance results?  *[text field]* | Copy + Paste any results or conclusions describing disparities or variances in autism services. | Include any results and conclusions regarding disparities or variances in autism services, even if the results are null. Please include point estimates and confidence intervals if available. Please use the notation “[insert quote here]” p. [insert page number here] [insert additional context here, if needed] |
| 13c | What were the main results shown in tables/figures that answered their research question(s)?  *[file upload]* | Create a file with all tables and figures presented by the authors that addressed the research question(s). | File upload. If these results cannot be captured in one snip/ screenshot, please paste them onto one Word document that you can then upload. |
| 14 | How do the authors define services?  *[text field]* | Describe and/or Copy + Paste how the authors define the services included in their study. | Include how services were defined (e.g., using procedure codes) and who made the choice for what qualified as a “service” and which services to include (e.g., study PI, clinician, etc.). Please use the notation “[insert quote here]” p. [insert page number here] [insert additional context here, if needed].  If not reported, write “not reported”. |
| 15 | What types of services were included in this study?  *[select all that apply]* | Based on the definitions provided in the codebook, please choose which service types best represent those studied in this article. | [ ] **Outpatient acute care**: when the patient does not need to stay at the clinic or hospital, including visits for otitis media or pharyngitis.  [ ] **Psychiatric service(s)**: any experience with a psychiatrist, in a psychiatric clinic or ward.  [ ] **Preventative care**: this includes well-child visits and routine developmental screening  [ ] **Emergency department**: when a patient is seen in the emergency department or an emergency room.  [ ] **Inpatient/hospitalization**: when a patient receives care through an extended stay at a hospital or clinic.  [ ] **Therapy services**: any intervention or therapeutic service to address development or symptoms, including speech and language therapy, occupational therapy, physical therapy, behavioral therapy, etc.  [ ] **Prescription medication**: use or prescription of medications or drugs.  [ ] **Over-the-counter medication**: use or access to medications or drugs that can be attained without a prescription.  [ ] **Foster care**: any patient receiving foster care services or within the foster care system.  [ ] **Diagnostic service(s)**: any services related to the ultimate clinical or educational diagnosis of a condition, disability, or disorder.  [ ] **Other care/service (please specify)**: select this option if the type of care or service included by the authors does not fall into the above categories. Please Copy+Paste the information the authors share about their included services and add additional context as needed. Please use the notation “[insert quote here]” p. [insert page number here] [insert additional context here, if needed] |
| 15a | What kind of therapy services were included in this study?  *[select all that apply]* | Based on the definitions provided in the codebook, please choose all types of therapies included in this article. | [ ] **Behavioral therapy**: typically administered by a behavioral therapist, a form of therapy that helps patients identify and change unhealthy behaviors or behaviors that are impeding their daily lives.  [ ] **Occupational therapy**: typically administered by an occupational therapist, a form of therapy that encourages rehabilitation through performing activities required in daily life to mitigate existing symptoms or conditions or recuperate from an injury.  [ ] **Physical therapy**: typically administered by a physical therapist, a form of therapy that treats ailments and injuries, and mitigates pain or discomfort due to deformities, symptoms, or specific conditions through physical methods like massage, heat therapy, and exercise.  [ ] **Speech and language therapy**: typically administered by a speech-language pathologist, a form of therapy that helps patients with communication (articulation, speech production, aphasia, social communication difficulties) and swallowing difficulties.  [ ] **Other (please specify)**: select this option if the type of therapy included by the authors does not fall into the above categories. Please Copy+Paste the information the authors share about their therapy services and add additional context as needed. Please use the notation “[insert quote here]” p. [insert page number here] [insert additional context here, if needed] |
| 16 | How was autism defined?  *[text field]* | Copy + Paste how the authors defined “autism” within their study sample. | This may include diagnostic codes, self-reports, EHR designation, assessment scores, probabilistic matching, etc. If the authors explain how they defined “autism” in multiple places, include all the information that will create a complete picture of what the authors intended. Please use the notation “[insert quote here]” p. [insert page number here] [insert additional context here, if needed]  If they do not report it, write “not reported” |
| 17 | How were conditions and/or diagnoses defined?  *[text field]* | Please describe how the authors define the diagnoses and/or conditions they are investigating. | Include any diagnostic codes and/or criteria used to define the diagnoses and conditions of interest.  If they do not report it, write “not reported” |
| 18 | What levels of factors or types of variables were evaluated to explore these variations or disparities in autism services?  *[select all that apply]* | Based on study’s methods, results, and conclusions, please select the factor types explored for variation or disparity in autism services based on the codebook definitions. | [ ] **Patient**: any variables related to the individual patient whose Medicaid claims are used, such as characteristics or demographics.  [ ] **Family/Caregiver**: any variables related to the family or caregiver of the patient whose Medicaid claims are used, such as characteristics or demographics.  [ ] **Provider**: any variables related to the provider of the patient whose Medicaid claims are used, such as demographics or discipline.  [ ] **Organizational and Regulatory (Systemic)**: any variables related to the system where the patient or Medicaid claim was, including laws/policies and administrative or visit structure.  [ ] **Local**: any variables related to the community where the patient is or the Medicaid claim was made, such as rurality and neighborhood poverty.  [ ] **Historical or Cultural**: any variables related to the historical or cultural climate where the patient is or the Medicaid claim was made, such as the COVID-19 pandemic or time/year-related trends.  [ ] **Unclear/Other (please describe)**: select this option if the type of factors/variables included by the authors do not fall into the above categories. Please Copy+Paste the information the authors share about their factors or variables and add additional context as needed. Please use the notation “[insert quote here]” p. [insert page number here] [insert additional context here, if needed] |
| 18a | Patient Level Factors  *[select all that apply]* | Please select all types of patient level factors or variables used by the authors to explore variations or disparities in autism services. If the patient factor or variable does not fit into one of the options based on the codebook definitions, please describe it in "Other". | Beyond descriptive demographic data…  [ ] **Age**: the patient’s age or age-based trends  [ ] **Biological sex**: the patient’s sex assigned at birth (not their gender identity) or sex-based differences  [ ] **Race and ethnicity**: the patient’s race, ethnicity, or combination of race and ethnicity.  [ ] **Language**: the patient's preferred, used/spoken, or household language(s).  [ ] **Socioeconomic status or income**: the patient’s access to resources, money, or a proxy measure like insurance dual-enrollment. If the study is assessing household income and the patient population is comprised of children, please select this response in the family/caregiver level factors instead of here.  [ ] **Generational/immigration status**: whether the patient is an immigrant or what generation they are within the U.S.  [ ] **Developmental/medical concerns-type-severity**: the patient’s developmental conditions, diagnoses, or disorders as well as any medical concerns including type of concern and severity of condition.  [ ] **Other (please describe)**: select this option if the type of patient factor/variable included by the authors do not fall into the above categories. Please Copy+Paste the information the authors share about their patient factors or variables and add additional context as needed. Please use the notation “[insert quote here]” p. [insert page number here] [insert additional context here, if needed] |
| 18b | Family/Caregiver Level Factors  *[select all that apply]* | Please select all types of family/caregiver level factors or variables used by the authors to explore variations or disparities in autism services. If the family/caregiver factor or variable does not fit into one of the options based on the codebook definitions, please describe it in "Other". | Beyond descriptive demographic data…  [ ] **Age**: the family member’s or caregiver’s age or age-based trends  [ ] **Biological sex**: the family member’s or caregiver’s sex assigned at birth (not their gender identity) or sex-based differences  [ ] **Race and ethnicity**: the family member’s or caregiver’s race, ethnicity, or combination of race and ethnicity.  [ ] **Language**: the family member’s or caregiver’s preferred, used/spoken, or household language(s).  [ ] **Socioeconomic status or income**: the family member’s or caregiver’s access to resources, money, or a proxy measure like insurance dual-enrollment.  [ ] **Caregiver education level**: the highest level of education completed by the family member or caregiver.  [ ] **Generational/immigration status**: whether the family member or caregivers is an immigrant or what generation they are within the U.S.  [ ] **Caregiver disability**: whether the family member or caregiver has a disability or developmental condition themselves, including severity or medical/developmental concerns.  [ ] **Other (please describe)**: select this option if the type of family or caregiver factor/variable included by the authors does not fall into the above categories. Please Copy+Paste the information the authors share about their family factors or variables and add additional context as needed. Please use the notation “[insert quote here]” p. [insert page number here] [insert additional context here, if needed] |
| 18c | Provider Level Factors  *[select all that apply]* | Please select all types of provider level factors or variables used by the authors to explore variations or disparities in autism services. If the provider factor or variable does not fit into one of the options based on the codebook definitions, please describe it in "Other". | Beyond descriptive demographic data…  [ ] **Training**: the type or amount of training completed by a provider during their educational experiences and once they started practicing.  [ ] **Biological sex**: the provider’s sex assigned at birth (not their gender identity) or sex-based differences  [ ] **Race and ethnicity**: the provider’s race, ethnicity, or combination of race and ethnicity.  [ ] **Language**: the provider’s preferred, used/spoken, or household language(s).  [ ] **Years of experience**: the amount of time or years that a provider has been practicing in their field or the medical system/clinic.  [ ] **Provider type/discipline**: the department or subfield or specialty of the provider (e.g., PT, OT, SLP, Pediatrics).  [ ] **Other (please describe)**: select this option if the type of provider factor/variable included by the authors does not fall into the above categories. Please Copy+Paste the information the authors share about their provider factors or variables and add additional context as needed. Please use the notation “[insert quote here]” p. [insert page number here] [insert additional context here, if needed] |
| 18d | Organization and Regulatory (Systemic) Level Factors  *[select all that apply]* | Please select all types of Organization and Regulatory (Systemic) level factors or variables used by the authors to explore variations or disparities in autism services. If the Organization and Regulatory (Systemic) factor or variable does not fit into one of the options based on the codebook definitions, please describe it in "Other". | Beyond descriptive demographic data…  [ ] **Administrative structure**: the composition and hierarchy of a system, including department and leadership teams/roles.  [ ] **State law/policy**: legislation governing a specific state or territory.  [ ] **Federal law/policy**: legislation governing the entire U.S.  [ ] **Staffing**: the staffing practices or amount of staff available in a system.  [ ] **Service structure**: whether a system has family-centered practices, home-based services, or education-based services, including the location of service visits and type.  [ ] **Other (please describe)**: select this option if the type of systemic factor/variable included by the authors does not fall into the above categories. Please Copy+Paste the information the authors share about their system factors or variables and add additional context as needed. Please use the notation “[insert quote here]” p. [insert page number here] [insert additional context here, if needed] |
| 18e | Local Level Factors  *[select all that apply]* | Please select all types of local or community level factors or variables used by the authors to explore variations or disparities in autism services. If the local or community factor or variable does not fit into one of the options based on the codebook definitions, please describe it in "Other". | Beyond descriptive demographic data…  [ ] **Economic factors**: including neighborhood or community poverty index, access to resource or socioeconomic access, average/median income, or availability of employment.  [ ] **Educational factors**: including access to schools and other educational systems (e.g., Head Start, Preschool Promise, technical programs, etc.) as well as associated entry criteria and waitlists.  [ ] **Health factors**: including access to medical systems and care as well as associated waitlists, general availability, and distance.  [ ] **Community factors**: including general community characteristics like rurality/urbanicity, specific state/county/locale/region/ site, frequency of events within that community, or general practices or culture within that community.  [ ] **Other (please describe)**: select this option if the type of local factor/variable included by the authors does not fall into the above categories. Please Copy+Paste the information the authors share about their local factors or variables and add additional context as needed. Please use the notation “[insert quote here]” p. [insert page number here] [insert additional context here, if needed] |
| 18f | Historical or Cultural Level Factors  *[select all that apply]* | Please select all types of historical or cultural level factors or variables used by the authors to explore variations or disparities in autism services. If the historical or cultural factor or variable does not fit into one of the options based on the codebook definitions, please describe it in "Other". | Beyond descriptive demographic data…  [ ] **Time Trends (Year/Dates)**: looking at a specific year, date, or range when services were delivered, including time-based trends.  [ ] **COVID-19**: exploring services during the pandemic.  [ ] **War periods/civil unrest**: exploring services during war periods or when there are protests, including the Black Lives Matter movement.  [ ] **Red-lining**: exploring the effects of historical community segmentation by homeowners, builders, and realtor systems.  [ ] **Tribal sovereignty rulings**: exploring the effects of Supreme Court or local court rulings about the right of Indigenous tribes to govern themselves.  [ ] **Other (please describe)**: select this option if the type of historical or cultural factor/variable included by the authors does not fall into the above categories. Please Copy+Paste the information the authors share about their historical factors or variables and add additional context as needed. Please use the notation “[insert quote here]” p. [insert page number here] [insert additional context here, if needed] |
| *Participant Information* | | | |
| 19 | What was the autistic study population(s) included in the study?  *[select all that apply]* | Based on how the authors describe their participants, select all applicable options for the ages of autistic participants. | [ ] **Autistic infants (0-2 years)**: infants up to 35 months of age  [ ] **Autistic young children (3-5 years)**: children 36 to 71 months of age  [ ] **Autistic children/young adults (6 - 21 years)**: patients 72 months (6 years) of age to 21 years old  [ ] **Autistic adults (>21 years)**: patients older than 21 years. |
| 20 | Did the study include any non-autistic populations?  *[select one]* | Indicate whether a population without an autism diagnosis was included in this study. If yes, describe what their role was. | [ ] **Yes**: select this choice if non-autistic people were included.  [ ] **No**: select this choice if only autistic patients were studied. |
| 20a | Non-autistic population(s)  *[select all that apply]* | Please specify the non-autistic population included in the study based on the codebook definitions. | [ ] **Providers**: a medical provider of any discipline (e.g., nurses, PCPs)  [ ] **Educators**: an educational provider or instructors that operates within the educational system (e.g., special education teacher)  [ ] **Policy administrators**: policymakers or administrative staff including department directors and leadership staff.  [ ] **Family**: including any parents, other caregivers, or siblings.  [ ] **People with other conditions (please specify)**: patients with other diagnosed developmental or medical conditions, disabilities, or disorders (e.g., Down Syndrome, ADHD, Cerebral Palsy, OCD, etc.)  [ ] **Multiple categories (please describe)**: participants with multiple roles (e.g., a family member that’s also a provider) or roles that seem to transcend a few categories (e.g., school-based special education provider). Please describe the participant role as specified by the authors.  [ ] **Other (please specify)**: select this option if the participant type or role does not fit into any of the above categories. Describe the participant type or role as specified by the authors. |
| 20b | Provider type(s)  *[select all that apply]* | For the providers included in this study, please specify their discipline based on how the authors describe them. | [ ] **Medical**: select this option if the providers are medical or clinical, work with outpatient, clinic or hospital-based services.  [ ] **Educational:** select this option if the providers are in educational or school-based setting and/or provide services through Part B Early Childhood Special Education.  [ ] **Not Specified:** select this option if the providers are not described as medical or educational, as well as if only some of the providers are described but not others. |
| 21 | Did the authors study an autistic population with comorbid conditions?  *[text field]* | Indicate whether the autistic population in this study had any comorbid conditions. If they do, please describe the comorbidities. | [ ] **Yes (please describe)**: select this choice is patient with both a diagnosis of autism and another condition were studied.  [ ] **No**: select this choice if only autistic patients were studied. |
| 22a | Autistic Participant Total n’s  *[text field]* | Please capture the total N of autistic participants as reported by the authors. | Just type in a number without using N or n (i.e., 3502). For multi-cohort, we would try to take the overall number across cohorts (this can be included as a function like “=23+45+56”). For longitudinal we would take baseline numbers for the first data collection period/point. |
| 22b | All Participant Total n's  *[matrix]* | Please capture the total N of all condition types as reported by the authors. | Only fill out the information that is provided (i.e., don’t enter “0” if there is no information). If the authors only report percentages out of a larger total sample size, please enter the “n” corresponding to a specific condition as a function based on the information provided.  For example, if the authors state: “Out of 200 total participants, 20% had autism, 25% had autism-ADHD, 15% had OCD, and 40% had no diagnoses” you should fill out the following information:  Conditions: Total n:  Autism-only = 0.2 * 200  Other: autism-ADHD = 0.25 * 200  Other: OCD = 0.15 * 200  Other: no diagnoses = 0.4 * 200  For multi-cohort, we would try to take the overall number across cohorts (this can be included as a function like “=23+45+56”). For longitudinal we would take baseline numbers for the first data collection period/point. |
| 23 | Participant demographics: Race-ethnicity  *[matrix]* | Enter the total n associated with each race and ethnicity category of the population included in this study; include all applicable options. | Only fill out the information that is provided (i.e., don’t enter “0” if there is no information). If the authors only report percentages out of a larger total sample size, please enter the “n” corresponding to a specific condition as a function based on the information provided as described in the clarifications for 22b above.  For multi-cohort, we would try to take the overall number across cohorts (this can be included as a function like “=23+45+56”). For longitudinal we would take baseline numbers for the first data collection period/point.  [ ] **American Indian or Alaska Native:** use this label for an indigenous person, a native American or Alaska Native, and people who belong to federally recognized tribal nations.  [ ] **Asian:** use this label for a person of Asian descent. Can be used with nationality combinations (i.e., Asian-American) or with specific country descriptions (i.e., Chinese or Chinese American).  [ ] **Black or African American:** use this label to describe people who are phenotypically described as “Black”, have African ancestry, or are from Africa.  [ ] **Hispanic or Latino:** use this label to describe people who use Spanish or are from Latin America, a Spanish-speaking country, or Spain. Can be used with nationality combinations (i.e., Hispanic-American) or with specific country descriptions (i.e., Mexican or Mexican-American)  [ ] **Native Hawaiian or Pacific Islander:** use this label to describe people who are native Hawaiian or are from any pacific island.  [ ] **White:** use this label to describe people who are phenotypically described as “white” or Caucasian.  [ ] **Multiracial (please describe):** use this label to describe people who have multiple racial or ethnic identities or combinations (i.e., Black and White or Asian Hispanic). Describe the population in the text box as described by the authors.  [ ] **Missing/Not Reported**: including when participants preferred not to respond or answer was not collected for some or all participants  [ ] **Other (please specify)**: select this option if the race or ethnicity category or combinations described by the authors do not fall cleanly into any of the above categories. If there are multiple “other” categories, separate your reported values and descriptions with a semicolon [;] |
| 24 | Participant biological sex  *[matrix]* | Describe the total n for each biological sex category of the population included in this study; include all applicable options. | Only fill out the information that is provided (i.e., don’t enter “0” if there is no information). If the authors only report percentages out of a larger total sample size, please enter the “n” corresponding to a specific condition as a function based on the information provided as described in the clarifications for 22b above.  For multi-cohort, we would try to take the overall number across cohorts (this can be included as a function like “=23+45+56”). For longitudinal we would take baseline numbers for the first data collection period/point.  [ ] **Male:** must be biological sex, not gender  [ ] **Female:** must be biological sex, not gender  [ ] **Other (please describe):** including “intersex”. Please describe the label used by the authors for a different biological sex. Also, use this category if the authors only report gender identity. If there are multiple “other” categories, separate your reported values and descriptions with a semicolon [;]  [ ] **Missing/Not Reported:** including when participants preferred not to respond or answer was not collected for some or all participants |
| 25 | Participant Age(s)  *[select all that apply]* | Select the age range of the population included in this study; select all applicable options. | [ ] Under 3  [ ] 3-5 years  [ ] 6-11 years  [ ] 12-18 years  [ ] 19-35 years  [ ] 36-55 years  [ ] 56 years and over |
| 26 | Participant Language(s)  *[matrix]* | Describe the total number of users included for the household, preferred, or used language(s) of the population included in this study; include all applicable options. Review the codebook clarifications for options listed. | Only fill out the information that is provided (i.e., don’t enter “0” if there is no information). If the authors only report percentages out of a larger total sample size, please enter the “n” corresponding to a specific condition as a function based on the information provided as described in the clarifications for 22b above.  [ ] **English**: select this option if the authors explicitly mention that the participants used this language (not just that materials were developed in this language).  [ ] **Spanish**: select this option if the authors explicitly mention that the participants used this language (not just that materials were developed in this language).  [ ] Chinese: including Mandarin, Cantonese, Simplified, and Traditional Chinese as well as any other regional dialects of China. Select this option if the authors explicitly mention that the participants used this language (not just that materials were developed in this language).  [ ] Tagalog: including Filipino. Select this option if the authors explicitly mention that the participants used this language (not just that materials were developed in this language).  [ ] **Vietnamese**: select this option if the authors explicitly mention that the participants used this language (not just that materials were developed in this language).  [ ] **Arabic**: select this option if the authors explicitly mention that the participants used this language (not just that materials were developed in this language).  [ ] **French**: this does not include French-Creole. Select this option if the authors explicitly mention that the participants used this language (not just that materials were developed in this language).  [ ] **Korean**: select this option if the authors explicitly mention that the participants used this language (not just that materials were developed in this language).  [ ] **Russian**: select this option if the authors explicitly mention that the participants used this language (not just that materials were developed in this language).  [ ] **Portuguese**: this does not include Brazilian Portuguese. Select this option if the authors explicitly mention that the participants used this language (not just that materials were developed in this language).  [ ] **Other (please specify)**: select this option if another language not listed above is used in the study (e.g., American Sign Language). Provide any additional context needed.  [ ] **Unsure**: select this option if the authors report some language information but do not connect it clearly to their participants. For example, the authors may state “study materials were developed in English and Spanish” but then do not tell you what the language breakdown of the participants was, meaning that it is unclear whether there were English and Spanish or English-only participants in the study.  [ ] **Not Reported**: select this option if the authors do not provide any language information about |
| 27 | Participant Income/Socioeconomic status | Select the income of the population included in this study; select all applicable options. Review the codebook clarifications for options listed. | [ ] **Proxy**: select this option if a proxy measure for income or socioeconomic status was used to assess participants or their households, including insurance status or eligibility and dual-enrollment status.  [ ] **Federal Poverty Line Measures**: select this option if the socioeconomic status of the individual participant or their household is described using the federal poverty line. (e.g., 200% below FPL)  [ ] **Other (please specify)**: select this option if the authors use another way to describe the participant’s or their household’s income or socioeconomic status like neighborhood/census-block poverty or a range of income values (e.g., 0-20,000 annual income).  [ ] **Not Reported**: select this option if the authors do not share any information about participant income or socioeconomic status. |
